# Update on Clinical Trial Endpoints in Gene Therapy Trials for Inherited Retinal Diseases

**DOI:** 10.3390/jcm13185512

**Published:** 2024-09-18

**Authors:** Jane M. Igoe, Byron L. Lam, Ninel Z. Gregori

**Affiliations:** 1 Department of Ophthalmology, Bascom Palmer Eye Institute, University of Miami Miller School of Medicine, Miami, FL 33136, USA; jane.igoe@gmail.com (J.M.I.); blam@med.miami.edu (B.L.L.); 2 Miami Veterans Administration Medical Center, Miami, FL 33125, USA

**Keywords:** clinical trials, efficacy, endpoint, gene therapy, inherited retinal disease, inherited retinal dystrophy, inherited retinal degeneration, hereditary retinal degeneration, outcome measures

## Abstract

Inherited retinal diseases (IRDs) encompass a wide spectrum of rare conditions characterized by diverse phenotypes associated with hundreds of genetic variations, often leading to progressive visual impairment and profound vision loss. Multiple natural history studies and clinical trials exploring gene therapy for various IRDs are ongoing. Outcomes for ophthalmic trials measure visual changes in three main categories—structural, functional, and patient-focused outcomes. Since IRDs may range from congenital with poor central vision from birth to affecting the peripheral retina initially and progressing insidiously with visual acuity affected late in the disease course, typical outcome measures such as central visual acuity and ocular coherence tomography (OCT) imaging of the macula may not provide adequate representation of therapeutic outcomes including alterations in disease course. Thus, alternative unique outcome measures are necessary to assess loss of peripheral vision, color vision, night vision, and contrast sensitivity in IRDs. These differences have complicated the assessment of clinical outcomes for IRD therapies, and the clinical trials for IRDs have had to design novel specialized endpoints to demonstrate treatment efficacy. As genetic engineering and gene therapy techniques continue to advance with growing investment from industry and accelerated approval tracks for orphan conditions, the clinical trials must continue to improve their assessments to demonstrate safety and efficacy of new gene therapies that aim to come to market. Here, we will provide an overview of the current gene therapy approaches, review various endpoints for measuring visual function, highlight those that are utilized in recent gene therapy trials, and provide an overview of stage 2 and 3 IRD trials through the second quarter of 2024.

## 1. Introduction

Inherited retinal diseases (IRD) are a heterogeneous group of degenerative diseases that often lead to severe vision loss and blindness [1], and they are associated with over 280 causative genes identified to date [2]. IRDs affect an estimated 184,000 to 438,000 people in the United States and 5.5 million people worldwide [3]. Globally, the five most common IRD genes identified by a systemic review published through December 2022 were *ABCA4* (12.9%), *USH2A* (6.8%), *RPGR* (2.7%), *EYS* (2.1%), and *RHO* (1.9%), thus autosomal recessive (AR) Stargardt disease, non-syndromic retinitis pigmentosa, and syndromic Usher syndrome predominate [4].

Despite their relatively low prevalence, IRDs have a substantial impact on both individuals and society. Many IRDs present very early in life, requiring a lifetime of medical care and significantly impacting the independence, productivity, mental health, and well-being of the individual and family as a whole [5,6]. In 2019, IRDs were estimated to cost between 13 and 32 billion dollars annually in the United States alone if healthcare costs, individual productivity loss, and the impact on caretakers are considered [5]. In some high-income countries, IRDs are a leading cause of blindness in the working-age population, even surpassing diabetic retinopathy (DR) [7,8]. The last decade has seen a surge in interest in IRD research, positioning IRDs as a frontier indication for gene therapy due to technological advances coupled with optimal conditions of the eye for such treatments. Technological advances such as exponential decreases in the cost of sequencing [9]; breakthroughs in genetic engineering techniques such as CRISPR (Clustered regularly interspaced short palindromic repeats)/Cas-based systems for genome editing [10,11,12]; and the development of recombinant viral vectors for greater precision and efficiency in transduction [13,14,15,16,17] have all laid the groundwork for IRDs to emerge at the cutting edge of gene therapy.

The eye is an excellent candidate for gene therapy, being a small, accessible, immunoprivileged organ, with relative isolation due to the blood–retina barrier [17]. The monogenic origins of many IRDs in non-replicating and differentiated cell populations of the retina further set IRDs apart as an ideal gene therapy target. In December 2017, the United States Food and Drug Administration (FDA) approved the first ocular gene therapy product, voretigene neparvovec-rzyl (Luxturna), for patients with genetic variants in both copies (alleles) of the *RPE65* gene, typically diagnosed as Leber congenital amaurosis (LCA) type 2 or severe early-onset retinitis pigmentosa (RP).The approval was based on the results of the phase 3 trial (NCT00999609) sponsored by Spark Therapeutics Inc. (Philadelphia, PA, USA), which utilized novel clinical endpoints suitable for the low-vision population. The main clinical endpoint was binocular performance on the multiluminance mobility test (MLMT) using a validated obstacle course under standardized dim luminance conditions, and a critical secondary endpoint of improvement in full-field light sensitivity threshold testing (FST), which measures the lowest illumination perceived over the entire visual field. The change in visual acuity (VA) averaged over both eyes (a secondary endpoint) was not statistically significant between the treatment and control arms, which set a new precedent of giving less weight to VA change in IRD treatment trials.

Demonstrating the efficacy of therapeutic interventions in IRD trials presents unique challenges due to phenotypic heterogeneity, slow progression of the conditions, preferential peripheral vision loss, and small recruitment pools of patients with often advanced vision loss. Conventional ophthalmic outcome measures can lack adequate sensitivity in IRD trials, and some phase 3 trials have failed to meet the primary endpoint despite demonstrating some statistically significant improvements, such as Biogen phase 3 STAR trial in choroideremia, which failed to show a 3-line visual acuity improvement (Biogen, Cambridge, MA, USA). IRD trials require novel, clinically meaningful endpoints tailored to the clinical characteristics of these unique subsets of patients. This review aims to provide an overview of contemporary gene therapy approaches for IRDs; describe the current primary, secondary, and exploratory outcome measures utilized in ongoing, terminated, or completed IRD gene therapy trials through July 2024; and summarize phase 2 and 3 gene therapy trials in IRD.

### 1.1. Methodology

A search for interventional clinical trials for IRDs was conducted in the federal database clinicaltrials.gov and the European Union (EU) Clinical Trial Registry. Trials were identified using the search terms listed below, from registry inception to present (July 2024). The following trial types were excluded from the summary provided by this review: observational, diagnostic, behavioral, pharmacological, supplement-based, cellular-based, device, withdrawn trials, long-term follow-up studies of phase 3 trials as well as trials without a provided endpoint, non-specific indications, or trials addressing secondary complications of IRDs. Furthermore, to focus the discussion on measurements of efficacy, phase 1 and 1/2 trials were excluded as well.

This process yielded a total of 24 gene therapy studies including 8 phase 2 trials, 7 phase 2/3 or 2b trials, and 9 phase 3 studies. These trials and their endpoints are described in Section 4. Efficacy Outcomes in Phase 2, 2/3 and Phase 3 Trials. Trials are identified throughout the text by their 8-digit national clinicaltrials.gov registry number, i.e., NCT number.

Search terms included variations on the following: inherited retinal disease, inherited retinal degeneration, genetic retinal, inherited retinal dystrophy, genetic retinal disease, IRD and retina, retinal dystrophy, retinal degeneration, inherited retinal dystrophy, hereditary retinal, inherited retinal dystrophy, inherited retinal degeneration, retinitis pigmentosa, Stargardt, Choroideremia, CHM, Usher, achromatopsia, XLRP, photoreceptor degeneration, rod-cone, cone-rod, Leber congenital amaurosis, LCA, congenital stationary night blindness, CSNB, Best disease, XLRS, X-linked juvenile retinoschisis, enhanced S-cone syndrome, Bardet-Biedl, Leber hereditary optic neuropathy, LHON, RVCL, Batten, Goldman-Favre, Refsum disease, neuronal ceroid lipofuscinoses, progressive cone, *ABCA4, USH2A, RPGR, RHO, EYS, REP1, CRB1, CEP290, CNGB3, RPE65, AIPL1, CRB1, MCDR1.*

### 1.2. Inherited Retinal Disease Classification

**Figure 1:**
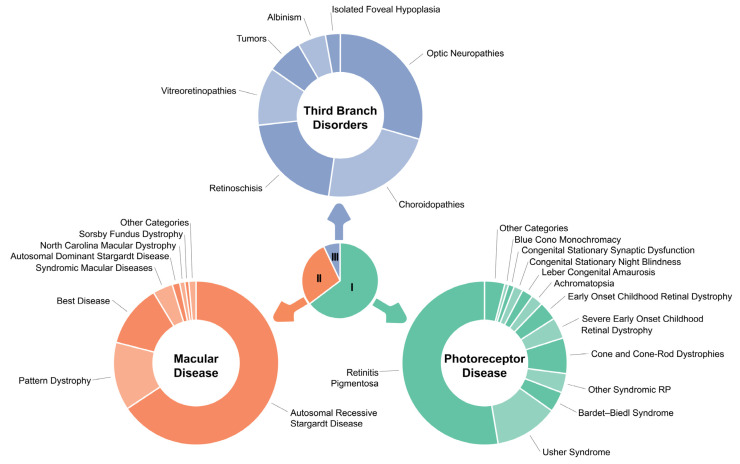
Graphical depiction of the distribution of 1000 consecutive probands among the larger diagnostic categories. Reprinted with permission from publisher—Stone et al. [19]. The center chart indicates the proportion of probands assigned to each of the three main branches of the classification system. The outer charts show the fraction of probands assigned to the larger diagnostics categories within each branch.

There are several systems for categorizing IRDs [18] based on criteria such as clinical characteristics, including stationary versus progressive conditions, syndromic versus non-syndromic manifestations, predominance of rod or cone involvement, timing of onset, anatomic presentations, or underlying etiologies. For this review, we will employ the clinical categories described by Stone et al. in the 2017 seminal publication based on genetic data from 1000 probands with non-syndromic and syndromic IRDs analyzed and classified by the Carver Nonprofit Genetic Testing Laboratory at the University of Iowa [19]. Namely, the IRD clinical categories are photoreceptor dystrophies (acquired, congenital, progressive, stationary rod–cone, cone, or cone–rod such as retinitis pigmentosa (RP), LCA, early childhood onset retinal dystrophy (ECORD), severe early childhood onset retinal dystrophy (SECORD), achromatopsia, blue cone monochromacy, congenital stationary night blindness, Usher and other syndromes), macular dystrophies (Stargardt, pattern dystrophy, North Carolina Macular dystrophy, Sorsby, Malattia Leventinese, syndromic macular dystrophies, Benign Fleck Retina), and third-branch disorders encompassing choroidopathies such as choroideremia and Gyrate atrophy, X-linked and recessive retinoschisis, optic neuropathies, tumors such as von Hippel Lindau and retinoblastoma, vitreoretinopathies such as Stickler and Familial Exudative Vitreoretinopathy, albinism, and isolated foveal hypoplasia (See Figure 1 and Table 1). Two thirds of IRDs are non-syndromic; however, over 80 forms of syndromic IRDs have been described and are associated with at least 200 different genes [20].

## 2. Genetic Therapy Approaches

### 2.1. Introduction

Traditionally IRDs have been untreatable except for supportive measures such as low-vision devices to maximize functional vision and treatment of secondary complications such as macular edema, cataracts, glaucoma, and retinal detachment. With advances in sequencing and genetic engineering, gene therapies have emerged as the leading strategy, addressing the molecular genetic underpinnings of IRDs through both gene specific and gene agnostic therapy approaches [21]. Briefly, these therapeutic strategies are described below.

### 2.2. Gene-Specific Therapeutic Strategies

This approach includes (i) gene augmentation with viral and non-viral delivery, (ii) genome editing, and (iii) RNA-based strategies. Gene augmentation or replacement is the process by which a wild-type (WT) or genetically optimized copy of the causative gene of an IRD is delivered to target cells, such as photoreceptors or retinal pigment epithelium (RPE), that are partially or completely deficient in the protein encoded by the gene of interest [17,22]. Gene replacement is best suited for recessive conditions addressing haploinsufficiency or loss of function genotypes, which comprise most IRDs [23]. Gene replacement vectors can be broken into viral or non-viral synthetic nanoparticles containing copies of a gene, with non-viral vectors still in the preclinical stage of development [23,24,25,26,27]. Adeno-associated viruses (AAVs) are the most well-studied viral vectors for IRD gene therapies [17,22] as borne out by voretigene neparvovec-rzyl FDA-approval for *RPE65*-associated retinal degeneration and utilization in completed and/or ongoing clinical trials for choroideremia, achromatopsia, X-linked and recessive RP, and LCA, amongst others. There are ongoing preclinical efforts to continue to optimize AAVs in retinal and ocular cell populations [13,28,29,30].

Despite AAVs’ transduction efficiency and acceptable safety profile [17], there are several important limitations to AAV-based gene augmentation approaches, including inability to address dominant gain-of-function mutations, dominant negative effects, intronic or splicing mutation [31], and AAVs’ limited cargo size of 4.7 kilobase pairs (kbp), eliminating their application for large genes such as *ABCA4, CEP290, USH2A, EYS* [30]. Thus, alternative therapeutic approaches such as genome editing, RNA editing and interference [32,33], and antisense oligonucleotides (ASO) are being explored in preclinical and clinical work [34,35,36,37,38,39].

Genome editing enables the highly precise modification, removal, and replacement of target host DNA sequences, halting of the expression of the mutant protein, and inducing of the expression of the WT proteins. There are several genome editing techniques, but CRISPR/Cas systems, which use sequence-specific guide RNA to target the host gene of interest and selectively correct or inactivate pathogenic sequences using endogenous mechanisms, have emerged as the most promising [40]. A phase 1/2 Brilliance clinical trial using a CRISPR/Cas system, delivered into photoreceptors via AAV5 injected into the subretinal space, was performed in patients with *CEP290*-associated LCA10 (NCT03872479) [41]. Three out of fourteen patients experienced clinically meaningful improvements in BCVA and two of the following endpoints: full field sensitivity test (FST), visual function navigation course (VFN), or the visual function quality of life (VFQ). Despite these positive data, the study terminated enrollment due to the very limited estimated population of patients in the U.S. and the company’s decision to seek a collaboration partner to continue the development of this gene therapy product. The most important risk of this approach is the introduction of unintended mutations at the genomic locations homologous to the target site, known as off-target editing, and the extent of risk requires further study and has been somewhat controversial [23,42,43]; however the Brilliance trial did not report any adverse events related to the CRISPR/cas intervention [44].

RNA editing strategies mimic a normal biological process that occurs in human cells and offer safety benefits over genome editing, as they do not permanently alter the host genome, and any potential off-target effects would be transient [23]. RNA editing is primarily suited to the treatment of IRDs caused by recessive mutations within larger genes which cannot be packaged into AAVs [23]. The technique uses sequence-specific guide RNA to selectively target host mRNA and recruit the endogenous RNA repair system, with adenosine deaminase acting on RNA (ADAR) to correct pathogenic mutations in the host mRNA, and is in preclinical stage of development [45,46]. The applications of RNA editing are currently limited by editing efficiency and potential off-target effects [23].

Antisense oligonucleotides (ASOs) are small 15–30-base single-stranded RNA or DNA sequences that can alter mRNA splicing, inhibit protein translation, and silence genes. Thus, ASOs may be applied to dominant IRDs and recessives genes with splicing defects, and they can be delivered into the eye via an intravitreal injection as they are able to penetrate the retina [47]. This approach has been explored in now-terminated phase 1/2 clinical trials involving an intronic mutation of *CEP290* and mutations in *USH2A* exon 13 sponsored by ProQR Therapeutics (Leiden, The Netherlands). Moreover, PYC Therapeutics, LLC (Australia), is exploring a peptide–oligonucleotide construct targeting patients with *PRPF31*-associated autosomal dominant (AD) RP to target the pre-mRNA of the *CNOT* gene that downregulates *PRPF31* expression in an attempt to increase the expression of splicing factor 31 and improve retinal function (NCT06455826, NCT05902962).

Furthermore, gene knockdown approaches via small interfering RNA (siRNA), which are double-stranded RNAs of 21–23 base pairs in length that trigger RNA interference (RNAi) and cause the breakdown of target mRNA, are being explored for autosomal dominant IRDs and are in the preclinical stages of development at the time of writing of this manuscript [48,49,50,51]; the technology, administered topically, is currently being tested in phase 2 trials for the treatment of neovascular macular degeneration, with early-phase trials planned for retinitis pigmentosa (NCT05637255, Sylentis, Madrid, Spain).

### 2.3. Gene-Agnostic Gene Therapy

Because IRDs are caused by hundreds of genes, it is important to develop gene-independent therapies, which may be applied regardless of the disease-causing gene to benefit a wider range of IRD patients. Gene therapy that may be applicable to multiple IRDs is commonly referred to as gene-agnostic gene therapy and includes modifier gene therapy and optogenetics. Gene-agnostic therapy is more accurately described as multiple-gene-targeted treatment, given that it is not possible that one treatment can benefit all IRDs.

Gene-agnostic modifier gene therapy is based on nuclear hormone receptor (NHR) genes, master gene regulators, which have the potential to restore homeostasis within target cells and to address multiple retinal diseases caused by multiple genes. Currently, Ocugen, Inc. (Malvern, PA, USA) is exploring an NHR gene, *NR2E3*, which regulates diverse physiological functions within the retina—such as photoreceptor development and maintenance, metabolism, phototransduction, inflammation, and cell survival networks—in RP (phase 3, NCT06388200), RP/LCA (phase 1/2, NCT05203939), AR Stargardt (phase 1/2, NCT05956626), and dry age-related macular degeneration (AMD) with geographic atrophy (phase 1/2 NCT06018558).

Rod-derived cone viability factor (RDCVF) is being explored in a multicentric phase 1/2 trial to assess the safety, tolerability as well as preliminary efficacy and quality of life following a single subretinal injection of SPVN06 (SparingVision, Paris, France), aiming to slow down central vision loss due to cone degeneration in patients with rod–cone dystrophy (RCD), caused by different genes (NCT05748873). The trial is recruiting adult patients with RCD due to a mutation in the *RHO*, *PDE6A*, or *PDE6B* genes.

Optogenetics represents an important gene-agnostic therapeutic approach for late-stage IRDs where there is already significant atrophy of photoreceptor (PR) layer. This approach inserts genes encoding photosensitive proteins to intact inner retinal cells, namely bipolar or retinal ganglion cells (RGCs), by delivering an opsin gene via a viral vector. Multiple companies are working with various opsins, such as channelrhodopsin-2, channelrhodopsin-CrimsonR, halorhodopsin, or multi-characteristic-opsin (MCO). The RESTORE trial (Nanoscope Therapeutics, Dallas, TX, USA) is testing sonpiretigene isteparvovec (MCO-010) delivered by intravitreal injection for treatment of end-stage retinitis pigmentosa (NCT04945772). MCO-010 is an AAV2 vector delivering multi-characteristic opsin into bipolar cells enabling the inner retina to be activated by ambient light across the visual spectrum. Following treatment with MCO-010, statistically significant improvements were seen on multiple measures of visual function including the primary outcome measure the change from baseline in vision-guided mobility using the Multi-Luminance Y-Mobility Test (MLYMT) at 52 weeks, as well as other key assessments including Multi-Luminance Shape Discrimination Test (MLSDT), near object recognition, and visual acuity by the computerized Landolt C scale. This approach to late-stage disease, where the photoreceptors may be degraded beyond the point of rescue, could help overcome some of the challenges posed by the phenotypic and genotypic diversity of IRDs, and may potentially benefit larger populations of individuals with these conditions. Other gene-agnostic approaches include cell therapy, neuroprotection, pharmacologic intervention, and rehabilitative or prosthetic approaches; however, these are beyond the scope of this review.

## 3. Endpoints in Inherited Retinal Disease

### 3.1. Introduction

To date, in addition to product safety, the primary outcome in published IRD trials has been a mean change from baseline in best corrected visual acuity (BCVA), microperimetry, and/or ambulatory navigation/mobility mazes (see Table 2) [52,53,54]. Secondary outcomes have included low-luminance visual acuity (LLVA), visual field, full-field light sensitivity threshold (FST), visual perception by quality of life (QoL) questionnaires, electroretinogram (ERG), microperimetry, fundus photography, nystagmus testing, central retinal thickness as measured by optical coherence tomography (OCT), pupillary light reflex response, and photophobia testing.

Applying for an investigational new drug or device (IND or IDE) requires submission of a detailed protocol specifying the primary and secondary endpoints to be used to assess the efficacy of the proposed intervention and to judge the overall outcomes of a trial. Due to the importance of these measures to the validity of the trial, the FDA provides general guidance on endpoint selection [55,56,57], as well as periodic specialty-specific guidance [58,59]. For phase 3 trials, an endpoint should ideally measure a direct clinical outcome, evaluating whether an intervention makes a patient feel or function better, or survive longer [55]; “survival” in the case of ophthalmic trials refers to forestalling blindness [60]. The FDA prefers functional endpoints to measure treatment effect [58]; however, surrogate measures of a direct clinical measure may be used when reasonably likely or expected to predict clinical benefit [56] by epidemiological, therapeutic, pathophysiologic, or other evidence [61]. Ultimately, approval of an IND requires the FDA to agree that the selected endpoints have been sufficiently validated for use.

Outcomes for ophthalmic trials measure changes in three main categories—structural, functional, and patient-focused outcomes. Since IRDs are either congenital or more insidious compared to conditions such as AMD or DR, and the visual acuity and the macula may be affected either very early or late in life, typical outcome measures such as central visual acuity and OCT imaging of the macula may not provide an adequate representation of therapeutic outcomes including alterations in disease course. Thus, alternative unique outcome measures are necessary to assess loss of peripheral vision, color vision, night vision, or contrast sensitivity in IRDs. These differences have complicated the assessment of clinical outcomes for IRD therapies, and the clinical trials for IRDs have had to design specialized functional and structural assessments to demonstrate treatment efficacy. Here, we will review these assessments, emphasizing which endpoints have been approved for use in IRD trials, as well as provide an overview of the current completed and ongoing phase 2 and 3 clinical trials in IRDs.

### 3.2. Functional Assessments

Best-corrected visual acuity, as well as static or kinetic perimetry, have been the “gold standards” for measuring efficacy in ophthalmology, and they have historically been preferred as primary endpoints by the FDA [58]. Visual performance consists of two components: visual function, meaning how well the basic visual system detects stimuli; and functional vision, or how well a person performs vision-dependent activities of daily living (ADLs) [62]. Functional assessments vary in their objectivity, ranging from objective measures to self-reports from patients. Visual function, particularly central visual function, consists of many components including visual acuity, luminance sensitivity, contrast sensitivity, field of vision, and color vision. Overall, visual function varies depending on environmental conditions and the task being performed. For example, visual acuity is crucial to pattern recognition and reading fine print, whereas the ability to distinguish an object against its background measured by contrast sensitivity is more important for mobility, postural stability, driving, and ability to perform ADLs [63]. Here, we will cover key endpoints for measuring these different facets of central vision.

#### 3.2.1. Visual Acuity

Visual acuity is measured in trials as best corrected visual acuity (BCVA), typically using the Early Treatment Diabetic Retinopathy Study (ETDRS) protocol, frequently assessing the proportion of patients with improvement or loss of ≥15 letters. This protocol uses standardized lighting and set-up, and a LogMAR chart to provide a calculated visual acuity score that can be used as a continuous variable for statistical analysis [58]. While ETDRS BCVA is widely used as a primary endpoint for most retinal pathologies, and has been used in many late-stage IRD trials (see Table 2), it loses sufficient sensitivity on both ends of the visual function, with a basement effect at the threshold of count fingers (CF) and a ceiling effect due to foveal sparing in IRDs such as RP, where central vision declines late in the disease course [59,64,65,66]. Despite its limitations, BCVA is still included in all IRD trials as a primary or secondary endpoint [67].

Alternative electronic methods for VA testing are available, such as the Freiburg visual acuity test (FrACT), which uses Landolt C optotypes on a computer monitor [68]. FrACT is well-validated [69,70,71] and has the significant benefits of being automated, self-administered, computer-based [72], and, importantly, it can measure clinical changes in VA down to hand motions (HM), surpassing the low-vision range of the ETDRS protocol, proving helpful in settings of very low vision [68], such as the phase 2b RESTORE trial testing the safety and efficacy of MCO-010 optogenetic therapy for the treatment of advanced RP (Nanoscope, NCT04945772). To participate in the study, patients had to have a BCVA worse than 1.9 LogMAR (20/1600 Snellen equivalent). Using the FrACT as the primary endpoint, RESTORE demonstrated preliminary results suggesting efficacy with a statistically significant improvement > 0.3 LogMar, i.e., 15 letters in 6 of 7 (86%) high-dose patients [73]. There are several app-based methods to test VA, but further validation studies are needed to determine the ability to translate their results to ETDRS or Snellen acuities [74,75,76,77].

Luminance conditions significantly impact VA, and measuring low-luminance visual acuity (LLVA) can assess this relationship. Most commonly, to perform LLVA a 2.0 log unit neutral density filter is placed in front of trial frames to reduce luminance during an otherwise standard ETDRS BCVA assessment. It can also be performed by lowering monitor luminance in a digital assessment of BCVA [60,65]. The difference between LLVA and standard BCVA is described as the luminance deficit (LLD).

The LLVA has great clinical value. In the early stages of some IRDs such as choroideremia and *RPGR*-associated RP, LLVA may decline before any detectable change in high-luminance BCVA [65], and it is also a better predictor of future acuity loss [60,65,78,79]. Furthermore, LLVA correlates well with quality of life (QoL) in RP [80] and is a better indicator of visual function than standard BCVA in AMD with GA [81,82,83,84]. The best applications of LLVA as an endpoint are thought to be rare maculopathies and rod–cone dystrophies [65]. Change in LLVA, specifically the proportion of patients with ≥15 letter improvement, was used as the primary endpoint in the phase 2/3 gene therapy trial (Beacon Therapeutics, former Applied Genetic Technologies Corporation, Alachua, FL, USA) for the treatment of X-linked RP (NCT04850118).

#### 3.2.2. Contrast Sensitivity

Contrast sensitivity (CS) is the ability to differentiate between minute differences in shading or patterns to distinguish objects from their background [85]. It has been shown that for every 1.0 logMAR loss of VA, the corresponding deficit in CS ranged from 0.22 logCS for cataract to 0.97 logCS for RP [85]. CS is widely used as a secondary endpoint [67], and the FDA has indicated that CS would be an acceptable primary endpoint if clinically and statistically significant differences were detectable at multiple spatial frequencies and cycles [58,59].

Pelli–Robson charts are the most common clinical assessments of CS, but these and other clinical tests are typically limited in their spatial frequency and stimulus contrast sampling. For clinical trials, standardized digital methods are preferred, and the quick contrast sensitivity function (qCSF) test has emerged as a reasonable potential outcome measure [86,87]. Rather than requiring hundreds of trials as with the standard CSF, the qCSF requires less than 50 trials lasting 3–5 min per eye by using a Bayesian adaptive framework to optimally sample both stimulus contrast and spatial frequency [88]. There are ongoing efforts to validate the qCSF in AMD [89,90], and it is being studied for use in patients with rod–cone dystrophy, Stargardt (STDG) and Best disease [91]. The qCSF has a better correlation with vision-related quality of life measures (VRQoL) than VA [90,92], and it has high sensitivity and test–retest repeatability [86,93]. Given these characteristics, qCSF has been recommended as an endpoint [86], and, in particular, a secondary endpoint in STDG trials [91].

The computer-based Ora Variable Contrast Flicker test (Ora-VCF) and the tablet-based Ora Contrast Sensitivity Test (Ora-CST) have both shown promise in AMD and DR as potential new assessments of CS. The Ora-VCF demonstrated superior sensitivity to CS in AMD compared to Pelli–Robson and other static CS testing methods [94,95,96]. These methods may find their application in IRD trials in the future.

#### 3.2.3. Perimetry and Microperimetry

Rod degeneration leads to a progressive loss of visual field (VF), thus the other gold standard for visual function outcomes is retinal sensitivity, measured by perimetry. For clinical trials, perimetry is automated due to greater reliability and consistency, and either static or kinetic perimetry is acceptable as a primary endpoint from a regulatory standpoint [23]. Common perimetry metrics include the mean sensitivity (MS) and mean deviation (MD) of visual field sensitivity, as compared to age-matched normal controls, expressed in decibels (dB).

A common form of perimetry is standard automated perimetry (SAP), which uses a white stimulus on a white background and primarily tests long- and mid-wavelength-sensitive cones [23]. Standard perimetry has been applied to clinical trials of RP, and all inheritance patterns of RP have intrinsic annual VF decline rates, with X-linked RP genotypes generally being higher [97]. High test–retest variability for SAP is widely acknowledged, especially with increasing visual field deficits, as was initially demonstrated in glaucoma [98]; furthermore, areas with the most vision loss and greatest need for close observation demonstrate the highest rates of variability, to the point where tests can become invalid due to the breadth of the confidence intervals [99]. It is therefore vital for detailed reporting in perimetry, including standard deviation and confidence intervals.

In IRDs, even within the same confirmed diagnosis as in RPGR-associated RP, some studies report statistically and clinically significant annual rates of decline in mean sensitivity, while others find that the minimum interval to calculate a statistically significant change is 2.5 years [100,101], similar to the findings in glaucoma patients, where a large cohort study found it would take between 2.1 and 3.3 years to detect a −2 dB per-year loss in the mean deviation of SAP [102].

Jacobson et al. developed a method for measuring dark-adapted rod retinal sensitivity, allowing for a direct comparison of rod-driven versus cone-driven perimetric responses [103]. This technique has been helpful in assessing retinal function, and dark-adapted perimetry has been included as an exploratory endpoint in clinical trials for inherited retinal disease.

Goldmann perimetry is no longer favored for perimetry due to the availability of automated test methods, but its test–retest variability in IRD patients is reportedly between 9–13% and 20–25% [104,105,106]. Automated Octopus kinetic perimetry has now replaced Goldmann perimetry, given that the methodology is standardized, and is included as an exploratory endpoint in some clinical trials.

Light-adapting or dark-adapting a patient before testing can help to isolate rod versus cone function, and chromatic perimetry can selectively assess the function of different cones by using monochromatic stimuli on different background colors [23,107,108,109].

Microperimetry (MP) has been an important new testing modality in perimetry that can evaluate retinal sensitivity when central vision and fixation are poor due to macular pathology. In MP, live-tracking of a fundus image adjusts for eye movement to maintain a consistent field of testing [110]. Despite the name, microperimetry uses a stimulus size comparable to SAP and tests up to 30 degrees from the fovea in mesopic (cones and rods function), scotopic (rod function), and even photopic (cone function) conditions [110]. Microperimetry has been used as a primary endpoint in the X-linked RPGR-associated RP phase 2/3 Xirius trial (NCT03116113) [111] and as the endpoint in interventional trials across numerous IRDs including choroideremia [112,113,114,115], LCA [116,117], AR Stargardt disease (STDG1) [118,119], X-linked retinoschisis [120], X-linked and other forms of RP [111], and achromatopsia [110]. The FDA criterion for significant change on MP is the proportion of study eyes with a mean change of ≥7 dB, with improvement from baseline at ≥5 pre-selected loci. Selecting custom points at the end of the scotoma has been discussed. MP has been reported to have strong test–retest reliability, and volumetric analyses like total volume (Vtot) have been proposed as highly sensitive metrics in RPGR-associated retinopathy [121]; a learning effect has been observed, so performance of two tests before acquiring a baseline for an interventional trial has been suggested [122].

There are, however, important limitations to MP as an endpoint. Firstly, methods vary widely across trials, with variation in devices, testing strategies, and reporting metrics. All of these factors complicate validation efforts. Rather than validating one MP protocol, some researchers have proposed that MP be standardized by treatment or indication [110]. Another drawback is that MP testing is lengthy and challenging for both patients and operators [110].

#### 3.2.4. Full-Field Stimulus Threshold (FST)

The full-threshold stimulus test (FST) has become an important endpoint for measuring residual vision when perimetry cannot be performed due to severe constriction of the VF or inadequate fixation due to nystagmus or severe blindness. The FST is a psychophysical measure of whole-field retinal light sensitivity, where an LED light is flashed in a full-field Ganzfield dome at varying intensities and a patient indicates the presence or absence of stimuli to establish the lowest perceivable level of luminance [123]. The instrument presents white, red, blue, or green flashes to test rod- and/or cone-based perception.

There are several important advantages of the FST. It uses standard ERG equipment, making it widely available, and it can quantify retinal sensitivity down to the level of light perception. Variations in the test can preferentially evaluate different subsets of photoreceptors. Dark-adapted FST can distinguish between cone and rod deficits [124], and light-adapted FST (LA-FST) can distinguish between different cone subpopulations using chromatic stimuli and backgrounds [35,125,126,127]. Originally developed for patients with LCA, FST is now widely used in IRD trials. It is often used in conjunction with the MLMT as it can extend the dynamic range for assessing retinal sensitivity due to the ceiling of the MLMT, which cannot assess visual function below a luminance of 1 lux [128,129]. Furthermore, the FST has been shown to correlate closely with the MLMT and has been suggested as a valuable alternative if the MLMT is not available [129]. For example, the FST was used as the primary endpoint in the 2023 approval of voretigene neparvovec-rzyl in Japan (NCT04516369) [123].

While the FST is a valuable tool for overcoming the challenges of low vision and nystagmus in testing visual function, it lacks clear applications beyond LCA and is not considered as a primary endpoint measure. It has been recommended to develop a consensus document establishing guidelines for the clinical application of the FST, similar to the ERG standardization guidelines developed by the International Society for Clinical Electroretinography (ISCEV) [124,127].

#### 3.2.5. Electrophysiology

The electroretinogram (ERG) is an important electrophysiological measure of retinal function that has long been used in the clinical diagnosis and monitoring of IRDs. ERG testing provides an objective measure of retinal function by recording the electrical response of the retina as a whole and component cell types by varying testing conditions and [103] stimuli. Jacobson et al. have shown patients can be adapted to scotopic or photopic conditions before testing to differentiate between rod and cone function, respectively [103]. Common variations in ERG tests include photopic and scotopic full-field (ffERG) to test overall rod, cone, and bipolar cell activity [108,109,110,111], pattern ERG to isolate retinal ganglion cell function [130,131,132,133] as well as multifocal ERG (mfERG) and focal (also known as foveal) ERG to examine the function of the central retina, valuable in macular dystrophies [67]. ERG testing parameters have been standardized by the International Society for Clinical Electrophysiology of Vision (ISCEV), which provides a valuable framework for standardization in other endpoint modalities such as FST [127].

ERG is not used as a primary endpoint in phase 3 IRD trials due to some important limitations. ERG correlates poorly with BCVA and VF and is typically severely reduced to nearly non-detectable levels in early stages of retinitis pigmentosa, despite the presence of good visual acuity and measurable visual fields. For instance, ERG responses are often extinguished in the early stages of many IRDs, such as LCA, when BCVA and perimetry are still measurable [134,135,136,137,138]. Testing may be difficult for patients to tolerate, and a high level of technical expertise and training is needed for both the administration and interpretation of tests, the availability of which is limited outside of academic research centers [67,129].

Certain IRDs with distinctive ERG findings have been proposed as strong candidates for the use of ERG as an outcome measure. For example, achromatopsia typically features greatly reduced photopic or cone-mediated response amplitudes with preserved scotopic or rod-mediated function. In pre-clinical models of ACHM, rescue with gene therapy interventions has been able to restore cone amplitudes in knock-out models [139,140,141], providing a powerful objective demonstration of functional improvement that could prove useful in clinical trials as well.

XLRS is another condition with a classic and distinct ERG presentation, but the value of ERG as an endpoint is less clear. The condition is characterized by a reduced or electronegative b-wave in the scotopic rod–cone response due to compromised cone to bipolar cell synapses, but with a largely preserved a wave. The ratio of the b to a wave has been shown to decline with time and with the severity of the mutation [142]. B-over-a-wave ratio has been proposed as promising endpoint candidate; however, a systemic review and a longitudinal study over 12 years [143,144] found minimal statistical difference in the b/a wave ratio, and instead found that BCVA proved to be a more sensitive outcome [143]. This may be related to the natural history of XLRS, which tends to progress rapidly in early childhood and then remains largely stable [145], so that while ERG is highly useful as a diagnostic tool, it may prove less useful as an endpoint.

#### 3.2.6. Color Vision Testing

Color vision deficits (CVDs) can be detected in the early stages of many IRDs, such as cone–rod dystrophies (CRD), achromatopsia, and other cone dysfunction conditions; it is also seen as late-stage rod–cone dystrophies (RCDs) progress to cone dysfunction. Measures of CVDs have not yet been used as a primary endpoint, given that efficacy signals have not been demonstrated in phase 1 and 2 clinical trials. The FDA has indicated they could be, provided there is a scoring system that can demonstrate statistically significant changes, such as the Farnsworth–Munsell 100 Hue test (FM 100) [58].

The FM 100 is considered the “gold standard” for testing CVDs. It is a psycho-technical test that requires a patient to arrange color plates by hue. Formerly a lengthy and cognitively stressful manual test [146], computerized versions of the FM-100 have cut testing time in half [147]. The Cambridge Color Test (CCT) is another computerized color vision test and has been studied in IRDs for its ability to track CVD changes over time (NCT01878032). A variation of the CCT, the trivector subtest, can measure the sensitivity of long-, medium-, and short-wavelength cones. It has been shown to have good test–retest reliability, and is validated for clinical and research use in healthy adults [148].

#### 3.2.7. Pupillometry

Pupillometry, or the measurement of the pupillary light reflex (PLR) in response to stimuli, is another functional test of value as an endpoint in the setting of advanced vision loss. The PLR is driven by image-forming rods and cones, as well as non-image-forming intrinsically photosensitive retinal ganglion cells (ipRGCs), and it remains intact even as vision approaches “no light perception” (NLP) and ERG is extinguished. Transient pupillary light reflex (TPLR) is obtained with a sequence of full-field red or blue stimuli of increasing luminance with the contralateral eye patched and is an objective technique to test the visual pathway, including the retina and the optic nerve. The test is convenient, non-invasive, and noncontact, and it can be used to complement other visual function tests [149,150]. Pupillometry is used rarely as a secondary outcome measure in IRD studies, as the criteria for meaningful efficacy change has not been determined.

Other variations in pupillometry include photopic or scotopic adaptation, and altering the wavelength of the light stimulus, known as chromatic pupil campimetry (CPC). CPC can provide spatial and temporal metrics for rod and cone function, as well as detect improvements in rod function in response to treatment with voretigene neparvovec-rzyl [151]. A recent study identified statistically significant differences in pupillary responses among different RP genotypes, as measured by the cone functional volume, a CPC-derived metric; in addition, statistically significant correlations between cone function and age were seen in some genotypes, such as *USH2A*-associated RP [152].

#### 3.2.8. Photoaversion Assessment

Photoaversion or photosensitivity often present as an early symptom of IRDs, in particular cone and cone–rod dystrophies, and they can manifest as the earliest symptom in cone dystrophies before vision loss occurs [153], or as a primary subjective symptom as in achromatopsia (ACHM). Despite the clinical importance of photophobia, particularly in ACHM, quantifying its severity and progression has proven challenging. Manually, light sensitivity thresholds can be measured by observing a patient’s reaction to light emitted from a halogen bulb and modulated with a rheostat [154,155,156]; however, for the purposed of clinical trials, automated measures are needed.

The automated ocular photosensitivity analyzer (OPA)devicewas developed at the Bascom Palmer Eye Institute (BPEI) as a subjective functional test of a patient’s visual photosensitivity threshold (VPT) [157]. The BPEI group developed a companion Visual Photosensitivity Questionnaire to evaluate the presence and severity of light aversion. Results from the assessments were reproducible, quantitative, and demonstrated good test-test reliability, making both tools promising endpoint candidates [157,158].

As a subjective symptom, photophobia has more commonly been measured via questionnaires, and research efforts to improve and validate these tools are underway. In the development of the Utah Photophobia Symptom Impact Scale (UPSIS-17), researchers compared the novel questionnaire to the Korean Photophobia Questionnaire (KUMC-8) and found that the UPSIS had better correlation with light sensitivity thresholds. Analysis with person–item maps identified several questions that could be removed without affecting the quality or validity of the questionnaire, and it was shortened from 17 to 12 (UPSIS-12) [159].

### 3.3. Structural Endpoints

#### 3.3.1. Introduction

Structural endpoints are surrogate anatomical biomarkers designed to substitute for clinical biomarkers [21]. They must be validated to demonstrate they correlate with and have predictive value for meaningful functional changes [61]. In IRD trials, structural endpoints are derived from increasingly sophisticated and high-resolution imaging techniques. In some cases, the resolution of retinal imaging modalities is at the cellular level, and structural changes can be observed prior to functional changes, as with adaptive optics imaging of cone density [160,161]. Cellular resolution is especially useful in IRDs as interventions are often intended to restore or preserve specific cell populations [162], and greater sensitivity could mitigate the challenges posed by the slow rates of retinal degeneration in IRDs [163].

Beyond endpoints, retinal imaging has many important applications in IRD trials including patient stratification, monitoring of treatment responses, tracking disease progression, and assessing safety profiles [163]. Below we outline the structural imaging modalities used in IRD trials currently.

#### 3.3.2. Optical Coherence Tomography (OCT)

OCT is a powerful imaging modality that can provide several important surrogate endpoints. High-resolution (HR) OCT has “near histology” resolution, providing both qualitative and quantitative assessments of outer retinal atrophy and PR loss [23], and can be either spectral domain OCT (SD-OCT) or swept-source OCT (SS-OCT) [23]. Of the two methods, SD-OCT is more common, having a lower cost and widespread presence in retinal practices. However, SS-OCT may become an important imaging modality for IRDs in the future. SS-OCT offers a significantly faster image acquisition rate which can limit artifacts with poor fixation. Additionally, SS-OCT can image the vitreous, retina, and choroid simultaneously due to its longer wavelength of light compared to SD-OCT [164].

OCT measures of macular thickness are widely used in diabetic macular edema (DME) and AMD trials and have a place in IRD trials; however, more important are measurements of outer retinal thickness, including photoreceptor layer. Outer retinal thickness has been shown to correlate with visual field sensitivity [165]. Disruption of the highly reflective inner segment ellipsoid zone layer (EZ; also known as the inner segment/outer segment [IS/OS] line) has been shown in many IRDs to correlate to a loss of retinal function, including retinal sensitivity and visual acuity, and is a marker for the progression of disease over time [166,167,168,169,170]. There are many ways to quantify EZ changes, but the measurement of EZ width (EZW) and EZ area (EZA) are the most well-studied [162] and are currently under evaluation as endpoints in RP, XLRP, LCA, Stargardt, and other IRDs [162,171,172,173,174,175]. Measuring EZ endpoints requires better automation of the retinal layer segmentation, and more sophisticated algorithms and machine learning tools are being developed in RP, *USH2A*-related RP, ARMD, and choroideremia [171,176,177,178].

OCT can also be used to measure photoreceptor loss by combining 3D information from various retinal layer parameters, including hypertransmission defects, contiguous RPE layer loss, and outer retinal atrophy, and it represents an active research area aiming to determine if OCT could be a more sensitive measure of atrophy than fundus autofluorescence [179,180,181,182].

#### 3.3.3. Fundus Autofluorescence (FAF)

FAF is a non-invasive imaging modality that can measure RPE and PR atrophy and dysfunction. FAF is currently the preferred method for measuring atrophy due to the excellent contrast and resolution it provides [60,183,184].

Progressive outer retinal atrophy is a hallmark of a majority of IRDs and is easily visualized using FAF [23]. High metabolic stress leads to the accumulation of lipofuscin, which produces hyperfluorescent signal and often precedes the development of atrophy, seen as areas of hypofluorescence [185,186]. Patterns on FAF can aid in IRD diagnosis [187], and they can also help visualize the leading and trailing edges of atrophy to allow for the calculation of the rate of atrophy progression, as was demonstrated in the OAKS and DERBY trials studying intravitreal (IVT) pegcetacoplan for the treatment of GA secondary to AMD (NCT03525613, NCT03525600) [162,188,189]. In particular, areas of definitely decreased autofluorescence (DDAF) and questionable decreased autofluorescence (QDAF) were quantified and used to monitor the progression of Stargardt disease. QDAF is considered a transition state between a healthy retina and later stages of the disease, and both DDAF and QDAF represent monitoring tools for interventional clinical trials that aim to slow disease progression [190].

#### 3.3.4. Adaptive Optics (AO)

Originally developed for astronomical observation to compensate for optical aberrations from the atmosphere, AO uses deformable mirrors to correct optical aberrations so images are only limited by diffraction, making imaging of the retina at the cellular level possible [191,192], with a resolution of approximately 2 μM sufficient for visualizing individual photoreceptors which are 2–5 μM in size [193]. By comparison, the resolution of standard commercial SD-OCT devices is 5–7 μM [194].

AO has been applied to many imaging modalities, and scanning laser ophthalmoscopy (SLO) in particular has gained traction as it can provide the best resolution and contrast [195]. There are currently no AO metrics acceptable as an outcome measure in phase 3 trials; however, AO is expected to become an important future endpoint to measure efficacy, as well as aid in patient selection [23,60,196].

AO-SLO techniques can provide numerous metrics of photoreceptor (PR) health, including cone density, cone spacing, and cone regularity [197]. The cellular detail provided by AO has an important implication for future outcome measures, especially in gene therapy trials where the therapeutic aim is to prevent PR cell loss or restore PR cell function. Illustrating its sensitivity as an endpoint, several studies have demonstrated significant PR loss and structural changes prior to major functional loss [160,161,198], as well as preservation of the cone mosaic following a therapeutic intervention [199].

AO imaging may also prove important to IRD trials in the future, as cellular-level imaging will help screen for patients who are still in the therapeutic window prior to irreversible target cell changes [193,200,201]. Studies describing the reliability and variability of cone structure and function are essential to validate AO-SLO-derived metrics for use as longitudinal outcome measures [202]. AO requires specialized instruments and training to perform and is not widely available. In IRDs, the goal would be to see if cone density decreases less quickly with treatment, which is difficult to assess given test and re-test variability. Unfortunately, AO is not possible to obtain in patients with poor fixation.

#### 3.3.5. Optical Coherence Tomography (OCTA)

OCTA is another relatively new imaging modality increasingly utilized as a secondary or exploratory endpoint, but much work is needed before it can be used as primary endpoint. OCTA provides high-resolution visualization of the vasculature and microvasculature in the retina and choroid by measuring the movement of red blood cells in multiple scans over time, delineating vessels from the static surrounding tissues [203].

There is a growing body of evidence characterizing vascular changes in different IRDs, as seen on OCTA. Research findings have been variable, but trends are emerging. In their 2023 review, Iovani et al. found that across 19 studies of OCTA findings in RP, there was consensus observing significant reductions in the superficial capillary plexus (SCP) and deep capillary plexus (DCP). Studies varied significantly in the characterization of the choriocapillaris foveal avascular zone (FAZ), with 2/3 demonstrating a significantly increased FAZ and 1/3 demonstrating a significantly decreased FAZ [203].

While OCTA shows promise for the clinical diagnosis and monitoring of disease progression in IRDs, its precise role must be refined and standardized before its metrics have utility as outcome measures. Further challenging OCTA as a potential endpoint is that the capture of quality images requires repeated scans, which can prove quite difficult in people with poor fixation or nystagmus common in IRDs [164]. SS-OCTA can mitigate this challenge but motion and other artifact types remain common, even with faster acquisition rates and tracking algorithm, [164]. Randomized prospective studies with longer follow-up and larger sample sizes are needed to further define the pathogenesis and natural history of IRDs before OCTA has potential as a primary endpoint [203].

#### 3.3.6. Composite Endpoints

There are currently no composite endpoints (CEPs) that are FDA-sanctioned as outcome measures in IRDs; however, they are a promising frontier for endpoint development. CEPs combine two or more distinct endpoints of interest, called component endpoints, into a single variable for statistical analysis [204]; they are appropriate in conditions where more than one outcome is clinically important and multiple outcomes are expected to be affected by treatment [56]. The component endpoints of the CEP should be of similar clinical significance to prevent inaccurate conclusions from being drawn [205,206].

CEPs offer several advantages over a single endpoint. They increase the incidence rate of the endpoint to allow for a more sensitive detection of potential differences between treatment groups [56,60,207] and improve the ability to assess the net effect of an intervention [207]. Practically speaking, the added sensitivity of outcomes could allow for an adequate statistical power of smaller and potentially shorter trials, requiring fewer resources and bringing treatments to market faster [208], which is critical for these devastating orphan diseases.

The continual advancement of imaging techniques coupled with a growing body of evidence connecting structure and function relationships in the retina make IRD indications prime for the development of CEPs that combine structure and functional endpoints [60]. In one study, a CEP was developed and validated to measure disease progression in *ABCA4*-associated Stargardt disease. This condition has vast phenotypic variation, with over 900 genetic variants identified as of 2017, and rates of functional and structural progression differ among variants, limiting the success of phase 1 and phase 2 trials [209]. The CEP analyzed the expanding transition zone combined with BCVA, questionably decreased autofluorescence, definitely decreased autofluorescence, loss of EZ, and loss of ELM by looking at the mean-to-standard deviation ratio. Variables were weighted and compared to individual measures, with all possible weighing combinations modeled to arrive at the most sensitive endpoint. Interestingly, the most sensitive endpoint was a CEP that excluded BCVA, which was found to have a low signal-to-noise ratio. Machine learning presents a potent tool for CEP development, and in similar work, some groups have used it to synthesize imaging and functional outputs in RP and LCA to predict localized treatment potential [210].

### 3.4. Patient-Focused Outcomes (PFOs)

#### 3.4.1. Introduction

Patient-focused outcomes are an increasingly important category of endpoints as they are direct measures of the clinical relevance of an intervention. They fall into two main categories—performance-based tests (PBTs) and patient-reported outcomes (PROs). PBTs are designed to assess functional vision such as mobility or the ability to execute activities of daily living (ADLs), whereas PROs are self- or guardian-reported outcomes of a patient’s quality of life (QoL), health-related QoL (HRQoL), vision-related QoL (VRQoL), and symptom management. The multi-luminance mobility test (MLMT), a PBT, is currently the only PFO to be an approved primary endpoint, as was demonstrated in the approval of voretigene neparvovec-rzyl [129,211].

In 2019, an expert panel of IRD specialists identified the development of better PFOs as a top priority for assessing the true clinical relevance of a therapeutic intervention, citing the need for standardized and validated IRD-specific questionnaires [21]. Similarly, the FDA has also recommended an increase in patient-focused outcomes and has provided guidance on patient-focused drug development tools such as questionnaires [212].

#### 3.4.2. Performance-Based Tests (PBTs)

The primary focus of PBTs in IRD trials centers on mobility, though endpoints assessing ADLs are also in use or development. Vision-guided mobility tests, or orientation and mobility (O&M) tests, are methods for assessing functional vision as it relates to the ability to navigate safely and efficiently in simulated real-life conditions. These endpoints provide valuable information about the clinical relevance of an intervention, as reduced mobility is well established to have an adverse impact on QoL [67].

The MLMT was developed for the voretigene neparvovec-rzyl trial [128], and has since been adopted for use in other trials. It consists of a physical maze with obstacles which patients navigate in standardized lighting conditions from 1 to 400 Lux. Trained masked graders review videos for accuracy and speed and issue a pass or fail grade [128]. The MLMT is limited in that it cannot assess the ability to see at light levels lower than 1 lux. The Ora Visual Navigation Course (Ora-VNC; Ora, Inc.) is a mobility test similar to the MLMT, and it is currently in use as an endpoint in multiple phase 2 and 2/3 IRD trials (NCT06333249) [213,214,215,216].

The Luminance Dependent Navigation Assessment (LDNA) is a newer functional assessment with improved sensitivity compared to the MLMT, capable of evaluating function across a wider range of light intensity from 0.04 to 500 Lux. The LDNA was developed by Ocugen, in collaboration with the FDA, for their phase 3 pivotal trial of OCU400 for the treatment of *RHO*-mediated and gene-agnostic RP (Ocugen, NCT 063882200). This innovation allowed for the inclusion of participants with early to advanced stages of disease [217].

Other assessments of function vision, including performance in ADLs and mobility, have been developed in both academic and industry settings [213,218,219]. The functional low-vision observer-rated assessment (FLORA) is a comprehensive assessment of functional vision designed for administration before and after implantation of the Argus II retinal prosthesis; it assesses mobility, ADLs, reading, and other measures of independence [220]. The Reading Performance Testing and the Radner Reading Charts are two common reading assessments and both have been used to evaluate functional reading vision in the setting of IRD [221,222,223]. The multiluminance shape discrimination test (MLSDT) assesses near object recognition and has been used as a key secondary endpoint in Nanoscope’s Phase 2b trial of MCO-010 for advanced RP (NCT04945772).

Virtual reality (VR) mobility assessments are increasingly studied as alternatives to maze-based or physical O&M tests [224,225,226] due to the advantages of being safer, more standardized, and offering unlimited course variability. Furthermore, they require less space and personnel training, and provide instant, objective grading [227]. VR is also promising for evaluating the performance of ADLs. A 2023 study was able to detect gradations of visual function in a group of patients with variable levels of vision with a VR assessment of ADLs [228].

#### 3.4.3. Patient-Reported Outcomes (PROs)

In addition to understanding how well a patient can perform ADLs, sensitive, reliable, and validated measures are needed to capture patients’ experiences and perspectives on improvement in their functioning. PROs are typically questionnaires used as screening tools and subjective measures of visual function, and they can be valuable in bridging the gap where technology is not yet available for objective measurement [229].

There are currently no FDA-approved PROs for use as a primary outcome measure. They are not required in trials, but regulatory guidance recommends their use, and they are common secondary endpoints. The NEI-VFQ is used widely throughout ophthalmic trials and is well validated [230]; however, it is not specific to IRDs. Similarly, the Impact of Vision Impairment questionnaire (IVI), and a variation for very low vision (VLV-IVI), have long-standing use; however, neither is IRD-specific [231].

Several IRD-specific PROs are available; however, overall, quality and standardization need to be improved. A 2017 review of IRD-specific questionnaires identified nine for RP, one for CSNB, and one for STDG [232]. The review, however, found that these questionnaires were limited by inadequate validation, small sample size, and narrow scope, with specificity limited to a small subset of IRDs. The researchers indicated a growing consensus that rather than focusing on just one aspect of vision, PROs should be a holistic evaluation of VRQoL, including areas such as activity limitation, mobility, and social and emotional well-being [232]. Somes example of recent questionnaires specific to a wide range or IRDs include the Michigan Retinal Degeneration Questionnaire (MRDQ) and Michigan Vision-Related Anxiety Questionnaire (MVAQ); both were developed in accordance with FDA guidance on PROs [212] and were validated for use in both adults and adolescents [229,233,234].

Another consideration with PROs is their use and applicability in pediatric populations, which are increasingly included in phase 1 and 2 IRD trials [21]. The Society for Health Economics and Outcomes Research (ISPOR) provides the following guidance: children 12–18 years old should self-report health status, children should be involved in developing questionnaires, and new PROs should be developed specific to age groups. For children under 12, proxy reports, referred to as “Observer reported outcome” assessments (ObsRO), should be considered [235]. A recent industry and academic collaboration developed and validated the ViSIO-PROs and companion ViSIO-ObsRO to evaluate vision-dependent ADLs in young patients with RP and LCA [236,237,238].

## 4. Efficacy Outcomes in Phase 2, 2/3, and 3 Trials (Table 2)

A review of primary endpoints for completed and ongoing phase 2, 2/3, and 3 trials in gene therapy through July 2024 revealed trials across eight indications: biallelic *RPE65*-associated retinal dystrophy, *CEP290*-mediated LCA10, *USH2A*-mediated RP, *RPGR*-mediated XLRP, RP (unspecified), Stargardt, *MT-ND4*-mediated LHON, and choroideremia. Endpoint selection varied between indications, and in some instances within parallel trials from the same sponsor when conducted in different countries (USA, Europe, Asia). A summary of the trials and their outcome measure may be found in Table 2.

There were two *RPE65*-associated retinal dystrophy trials for the approval of voretigene neparvovec-ryzl: the US and EU-based trial for original approval (NCT00999609), and a subsequent trial conducted in Japan with commercial approval granted in 2023 (NCT04516369). The primary endpoint for the former trial was the novel mobility test, the MLMT. Trial data found a close correlation of the MLMT and the achromatic FST scores averaged across two eyes, and the FST was subsequently used as the primary outcome for the approval in Japan [123,127,129].

Two phase 2/3 trials tested the efficacy of sepofarsen (QR-110), an RNA anti-sense oligonucleotide (AON), targeting the c.2991+1655A>G variant in *CEP290*-mediated LCA10 (ProQR/NCT03913143). The ILLUMINATE trial tested serial intravitreal injections spaced between 3 and 6 months. The BRIGHTEN trial (NCT04855045), for children under the age of 8, was an open-label dose-escalating study with a double-masked arm comparing two different doses. Sepofarsen was well-tolerated; however, it did not meet the primary endpoint of the mean change in BCVA from baseline compared to sham. In post hoc analysis, however, sepofarsen-treated eyes saw a mean change in BCVA of −0.12 logMAR, an improvement not found in the fellow eye. Similar improvements were seen in critical secondary endpoints such as the FST and mobility [239].

Three additional trials from ProQR tested the RNA AON ultevursen for the treatment of *USH2A* exon13-mediated RP. Two were phase 2/3 trials that targeted different study populations—the SIRIUS trial (NCT05158296) for advanced vision loss, and the CELESTE trial (NCT05176717) for early to moderate vision loss. The primary endpoint for SIRIUS was the mean change from baseline in BCVA at month 18, and for CELESTE it was the mean change in retinal sensitivity as measured by static perimetry. In addition, the phase 2 open-label extension study HELIA (NCT05085964) had several secondary endpoints to evaluate efficacy, including BCVA, LLVA, EZA/EZW (on OCT), static perimetry, and microperimetry. In 2022, the ultevursen program was downsized to just the SIRIUS trial and then terminated [239], and in late 2023 ProQR sold both ultevursen and sepofarsen to Thea Laboratories.

There are three ongoing phase 3 protocols testing bilateral subretinal botaretigene sparoparvovec for the treatment of RPGR-associated XLRP (Janssen backed MeiraGTx). Two protocols are US-based (NCT04671433, NCT04794101) with the vision-guided mobility assessment (VMA) as the primary outcome. The third trial was Japan-based, and the outcome measure selected was the change from baseline in Mean Retinal Sensitivity Within the Central 10 Degrees (MRS10) in static perimetry (NCT05926583). These trials have surpassed enrollment goals, and an extension study has been initiated. No results have been reported; however, the company anticipates filing a Biologics License Application (BLA) with the FDA in 2024 [240].

A phase 2/3 trial, VISTA (Beacon Therapeutics/NCT04850118), is testing AGTC-501 for the treatment of RPGR-associated XLRP, following positive results from the phase 2 SKYLINE trial (NCT006333249) where 5 of 8 subjects had an improvement in retinal sensitivity on microperimetry [241]. Recruitment for VISTA is ongoing.

Recruitment is active for a phase 3 trial testing the efficacy OCU400-301 for the treatment of *RHO*-associated RP and gene-agnostic RP (Ocugen, NCT 063882200). The trial aims to enroll 150 participants, the first of which was dosed in June 2024. The primary endpoint is the proportion of treated versus untreated participants to achieve an improvement of at least 2 Lux levels on the LDNA [242].

Two phase 2 optogenetic trials from Nanoscope investigated the efficacy of MCO-010. The first trial was the phase 2b RESTORE trial for advanced unspecified RP (NCT04945772). The RESTORE trial met the primary endpoint of mean improvements in BCVA, as measured by Freiburg testing, demonstrating statistically significant improvement exceeding the clinically significant 0.3 LogMAR threshold (15 letters) [73]. The results from the phase 2 STARLIGHT trial for Stargardt dystrophy (NCT054417126) were positive as well, demonstrating clinically meaningful improvement in BCVA and a 3db gain in mean retinal sensitivity on the octopus VF testing [243]. Nanoscope has announced plans to submit a BLA for MCO-010 in the second half of 2024.

Three phase 3 trials from GenSight Biologics investigated the treatment of LHON with intravitreal lenadogene nolparvovec (trade name Lumevoq). In the first two trials, REVERSE and RESCUE, monocular treatment was administered in acute or chronic LHON, respectively (NCT02652780/NCT02652767), and in the third trial REFLECT, treatment was administered bilaterally (NCT05244304). All three trials saw statistically significant improvements in BCVA from nadir defined as worst BCVA encountered in the study, but the primary endpoints were not met. Interestingly, improvement in the contralateral eye was seen with unilateral treatment, diminishing the statistical significance of the difference in BCVA between the active- and placebo-treated eyes [244]. Lumevoq has been made available in select countries through early access programs (EAPs), and in March of 2024 GenSight reported real-world increases in BCVA of +18 and +23 ETDRS letters for unilateral versus bilateral treatment, respectively [245].

Two additional studies for LHON tested Neuropth’s rAAV-ND4 (NR082) via a single unilateral intravitreal injection. The first study was initiated by the Huazhong University of Science and Technology (NCT03153293), enrolled 40 subjects, and was completed in 2020. The treated eye was compared to the untreated fellow eye, and similar to the REVERSE and RESCUE trials, statistically significant improvements from baseline were seen in both eyes at the 12 month endpoint; however, there was no statistical difference between the two eyes and the study did not meet the primary endpoint [246]. In their discussion of the contralateral effect of treatment, authors postulate transport of the vector via the optic nerve across the chiasm to the fellow nerve, citing an animal study where such transport was seen following intravitreally injected fluorescein gold; however, the mechanism of transport was not understood [247]. The second study is the phase 2/3 GOLD trial (NCT04912843). NR082 received an IND from the FDA in 2022 and was the first Chinese-developed AAV product to receive an orphan drug designation. Enrollment in the GOLD trial was completed in China in 2023, and the first US sites were initiated in April 2023 [248]. Results have yet to be reported.

Three phase 2 academic trials studied the efficacy of a single subretinal injection of an AAV2 vector of *REP1* for the treatment of choroideremia. All three trials used the mean change in BCVA as the primary endpoint. Two smaller trials at the Bascom Palmer Eye Institute (NCT025531535) and the University of Tubingen (NCT02671539) enrolled six subjects each. Both studies found that vision could be maintained, and in some cases could achieve sustained improvement following treatment and that treatment was well-tolerated [113,249]. A larger trial of 40 subjects at Moorfields Eye Hospital (NCT02407678) found no signal of efficacy over the 24-month assessment, and a statistically significant decrease in visual fields was seen in treated eyes, which researchers postulate was related to surgery [250].

Finally, an industry-funded program was launched to develop an AAV2-REP1 vector for treating choroideremia. After positive results from the phase 2 GEMINI trial [251], the phase 3 STAR trial (Biogen, NCT03496012) commenced in 2017, followed shortly after by SOLSTICE, a long-term observational study of AAV2-REP1-treated patients from several trials (NCT03496012, NCT03507686, NCT02671539, NCT01461213, NCT02077361). Notably, the STAR trial failed to meet the primary endpoint, which was the proportion of patients to achieve ≥15 letters at 12 months. This trial has been of particular interest to the IRD community, as it underscores the risks of suboptimal endpoint selection, leading to trial failure [252]. Despite failing to meet primary and key secondary endpoints [253], four individuals gained ≥15 letters in the high-dose groups compared to zero in the control, and 14% and 18% of the respective high-dose and low-dose groups gained ≥10 ETDRS letters compared to just 2% in the control arm [254]. Authors of the trial publication noted that optimizing inclusion and exclusion criteria and selecting different endpoints would improve the evaluation of the trial outcomes [254].

## 5. Conclusions and Future Directions

In summary, the endpoints utilized in IRD gene therapy trials include functional, structural, and patient-focused outcome measures, and should be carefully tailored to specific intervention, clinical characteristics of the disease, and the level of visual impairment. One pragmatic strategy for selecting outcomes and endpoints in an IRD trial, proposed by Taylor et al., is to group IRD conditions by clinical presentation and primary pathophysiology, such as rod–cone degeneration, cone–rod degeneration, and macular degeneration, and to develop a framework based on these criteria as a starting point for endpoint selection [64]. Sensitive, feasible, and validated outcome measures are crucial to the validity and success of the trials and should remain an area for innovation and a chief concern in IRD trial design.

Looking forward, there is likely to be an increasing range of validated and FDA-accepted outcome measures to choose from to achieve the best “fit for purpose” in IRD trials based on the specifics of the disease and interventions being studied.

Clinical factors that could influence outcome selection include disease pathophysiology, mechanism of action of the intervention, anticipated therapeutic benefit, predominant cell types or anatomical structures affected, target therapeutic window, and corresponding clinical features based on the disease natural history. Practical considerations include accessibility of testing devices; feasibility and tolerability of the testing modality; and whether the endpoint is accepted by regulatory bodies as a valid outcome measure.

In the short term, there are several functional endpoints that may become more prevalent in trials, possibly as an outcome measure. There will likely be a movement away from the MLMT to more sensitive mobility assessments like the LDNA, now FDA-approved as the outcome measure for pivotal trials. The FrACT has proven a valuable endpoint in low-vision settings, given its ability to quantify lower levels of central vision, needing less space, no special equipment, and simpler personnel training compared to the ETDRS protocol. Although widely in use as a secondary endpoint, the FST is not currently accepted by the FDA as a primary endpoint; however, that may change in the future for some IRDs. The FST is highly correlated with the MLMT, and the ability to quantify retinal sensitivity with advanced vision loss where perimetry and microperimetry may no longer be sensitive is essential. Further validation and standardization are needed, however. With new automated measures of photophobia designed, it is possible that trials could measure symptomatic improvement in relevant conditions like achromatopsia.

Structural endpoints are also gaining momentum with the recent acceptance of atrophy measured by FAF as an outcome measure in AMD trials. There are ongoing research efforts to automate and standardize the quantification of EZ measurements by OCT. AO-SLO may also become more prevalent in the future, given its higher sensitivity and ability to image not just the presence or absence of photoreceptors, but also the degree of degeneration and integrity of organization. However, increased accessibility and validation studies are needed. Despite its growing importance and technological advances, imaging can still prove challenging in patients with very low vision, where fixation and nystagmus can hamper image acquisition and lead to artifacts, particularly in modalities requiring repeated scans like OCT-A.

Patient-focused outcomes remain invaluable as “real world” measures of clinical benefit and help anchor the focus of trials on discernable benefits to how a patient feels, functions, or maintains their vision. Well-designed and validated questionnaires for people of all cognitive abilities and ages are needed to understand and quantify the patient experience and provide insight into the true clinical benefit of an intervention. The recent development and validation of virtual reality (VR) assessments of functional vision suggest a possible impending shift toward VR performance-based testing as an efficient, widely accessible, and standardized way to directly measure functional vision.

Finally, with the increasing computing power and capabilities of machine learning, composite endpoints could prove a significant advancement in IRD trials by facilitating shorter, smaller, and more efficient trials capable of providing a more holistic and sensitive measure of the effect of an intervention.

## Figures and Tables

**Table 1:** Inherited retinal disease categories. Adopted from Stone et al. [19].

I—Photoreceptor Disease
A—Isolated
1—Acquired/Progressive
a—Retinitis Pigmentosa
i—X-linked
ii—Autosomal Dominant
iii—Autosomal Recessive
iv—Other Multiplex
b—Cone and Cone Rod Dystrophy
i—X-linked
ii—Autosomal Dominant
iii—Autosomal Recessive
iv—Other Multiplex
2—Congenital/Stationary
a—LCA
b—SECORD
c—ECORD
d—Achromatopsia (Congenital Stationary Cone Dysfunction)
e—Blue Cone Monochromacy
f—Congenital Stationary Night Blindness
i—X-linked
ii—Autosomal Dominant
iii—Autosomal Recessive with normal fundus
iv—Enhanced S-cone Syndrome
v—Fundus Albipunctatus
vi—Oguchi Disease
g—Congenital Stationary Synaptic Dysfunction
h—Delayed Retinal Maturation
B—Syndromic
1—Usher Syndrome
a—Type I
b—Type II
c—Type III
2—Bardet-Biedl Syndrome
3—Neuronal Ceroid Lipofuscinosis
4—Senior-Loken Syndrome
5—Joubert Syndrome
6—Microcephaly Congenital Lymphedema and Chorioretinopathy
7—Retinitis Pigmentosa with Ataxia
8—Peroxisomal Biogenesis Disorders
9—Cohen Syndrome
**II—Macular Diseases**
A—Autosomal Recessive Stargardt Disease
B—Best Disease
C—Pattern Dystrophy
D—Autosomal Dominant Stargardt Disease
E—Sorsby Fundus Dystrophy
F—Malattia Leventinese
G—North Carolina Macular Dystrophy
H—Syndromic Macular Diseases
1—Macular Dystrophy, Diabetes and Deafness
2—Pseudoxanthoma Elasticum
3—Homocystinuria with Macular Atrophy
4—Spinocerebellar Atrophy
I—Benign Fleck Retina
**III—Third Branch Disorders**
A—Choroidopathies
1—Choroideremia
2—Gyrate Atrophy
3—Late Onset Retinal Dystrophy
4—Nummular Choroidal Atrophy
5—Helicoid Peripapillary Chorioretinal Degeneration
B—Retinoschisis
1—X-linked
2—Recessive
C—Optic Neuropathies
1—Non-syndromic
a—Autosomal Dominant
b—Autosomal Recessive
c—Leber Hereditary Optic Neuropathy
2—Syndromic
a—Wolfram Syndrome
b—Hearing Loss
D—Tumors
1—von Hippel Lindau
2—Retinoblastoma
3—Tuberous Sclerosis
4—Gardner Syndrome
E—Vitreoretinopathies
1—Stickler Syndrome
2—Familial Exudative Vitreoretinopathy
a—Norrie Disease
b—Autosomal Dominant
3—AD Neovascular Inflammatory Vitreoretinopathy
4—Wagner Disease (Erosive Vitreoretinopathy)
5—Knobloch Syndrome
6—Heritable Vascular Tortuosity
a—Autosomal Dominant Retinal Vascular Tortuosity
b—Cerebroretinal Vasculopathy
c—Fascioscapulohumeral Dystrophy
F—Albinism
1—X-linked Ocular Albinism
2—Oculocutaneous Albinism
a—Nonsyndromic
b—Hermansky Pudlak
c—Chediak Higashi
G—Isolated Foveal Hypoplasia

**Table 2:** Primary endpoints in phase 2, 2/3, and 3 gene therapy trials for inherited retinal diseases.

Indication	NCT Number	Status	Phase	Interventions	Sponsor	Retinal Function			Functional Vision
						BCVA	VF	FST	Mobility
Biallelic *RPE65*- associated dystrophy	NCT00999609	FDA Approved	3	voretigene neparvovec-rzyl (subretinal OU)	Spark Therapeutics				**X** MLMT
Biallelic *RPE65*- associated dystrophy	NCT04516369 *	Japan Approved	3	voretigene neparvovec-rzyl (subretinal OU)	Novartis			**X**	
LCA10 *CEP290-* mediated	NCT03913143 NCT04855045 ILLUMINATE/BRIGHTEN	ACTIVE Did not meet 1° Endpoint	2/3	sepofarsen (QR-110) (monocular IVT)	ProQR	**X**			
RP (*USH2A*- Mediated)	NCT05085964 HELIA	TERMINATED For business reasons	2	ultevursen (QR-421a) (Monocular or OU IVT)	ProQR	**X (2°)** Standard & LLVA			
RP (*USH2A*- Mediated)	NCT05158296 SIRIUS	TERMINATED	2/3	ultevursen (QR-421a) (Monocular IVT)	ProQR/Thea	**X**			
RP (*USH2A*- Mediated)	NCT05176717 CELESTE	TERMINATED For business reasons	2/3	ultevursen (QR-421a) (Monocular IVT)	ProQR		**X**		
XLRP *(RPGR*-mediated)	NCT04671433 MGT-RPGR-021	ACTIVE Not Recruiting	3	botaretigene sparoparvovec AAV5-hRKp.RPGR (subretinal OU)	MeiraGTx/ Janssen				**X** VMA
XLRP *(RPGR*-mediated)	NCT04794101 MGT-RPGR-022	ACTIVE Not recruiting	3	botaretigene sparoparvovec AAV5-hRKp.RPGR (subretinal OU)	MeiraGTx/ Janssen				**X** VMA
XLRP *(RPGR*-mediated)	NCT05926583 *	RECRUITING	3	botaretigene sparoparvovec AAV5-hRKp.RPGR (subretinal OU)	Janssen		**X** MSA-10		
XLRP *(RPGR*-mediated)	NCT06275620 DAWN	ENROLLING By invitation	2	AGTC-501 rAAV2tYF-GRK1-hRPGRco (subretinal injection)	Beacon/former AGTC	**X (2°)**	**X (2°)** MP, MAIA		
XLRP *(RPGR*-mediated)	NCT06333249 SKYLINE	ACTIVE Not recruiting	2	AGTC-501 rAAV2tYF-GRK1-hRPGRco (subretinal injection)	Beacon/former AGTC		**X** MP, MAIA		
XLRP *(RPGR*-mediated)	NCT04850118 VISTA	RECRUITING	2/3	AGTC-501 rAAV2tYF-GRK1-hRPGRco (subretinal injection)	Beacon/former AGTC	**X** LLVA			
XLRP (*RPGR*-mediated)	NCT03116113 XIRIUS	COMPLETED Did not meet 1° endpoint	1/2/3	cotoretigene toliparvovec AAV8-RPGR BIIB112 (Monocular, subretinal)	Biogen (formerly NightstaRx)		**X** MP, MAIA		
RP (*RHO*-mediated and gene-agnostic RP)	NCT06388200 liMeliGhT	RECRUITING	3	OCU400-301 (Bilateral Subretinal Injection)	Ocugen	**X (2°)** LLVA			**X** LDNA
RP (unspecified)	NCT04945772 RESTORE	COMPLETED Met primary endpoint	2b	MCO-010 (Monocular IVT)	Nanoscope	**X** (FrACT)			**X (2°)** MLYMT
Stargardt	NCT05417126 STARLIGHT	COMPLETED Phase 3 trial in planning	2	MCO-010 (Monocular IVT)	Nanoscope	**X (2°)**			**X** MLMT
LHON (*MT-ND4* associated)	NCT02652780 REVERSE	COMPLETED Did not meet 1° endpoint	3	lenadogene nolparvovec (Monocular IVT)	GenSight Biologics	**X**			
LHON (*MT-ND4*- associated)	NCT02652767 RESCUE	COMPLETED Did not meet 1° endpoint	3	lenadogene nolparvovec (Monocular IVT)	GenSight Biologics	**X**			
LHON (*MT-ND4*- associated)	NCT03293524 REFLECT	ACTIVE Not recruiting	3	lenadogene nolparvovec (IVT OU)	GenSight Biologics	**X**			
LHON (*MT-ND4*- associated)	NCT03153293 **	ACTIVE Not recruiting	2/3	rAAV2-ND4 (Monocular IVT)	Huazhong University of Science and Technology	**X**			
LHON (*MT-ND4*- associated)	NCT04912843 GOLD	RECRUITING	2/3	NR082 rAAV2-ND4 (Monocular IVT)	Wuhan Neurophth Biotechnology	**X**			
Choroideremia	NCT02553135	COMPLETED	2	AAV2-REP1 (Monocular, subretinal)	University of Miami, Bascom Palmer Eye Institute	**X**			
Choroideremia	NCT02407678 REGENERATE	Did not meet 1° endpoint	2	AAV2-REP1 (Monocular, subretinal)	University of Oxford, Moorfields Eye Hospital	**X**			
Choroideremia	NCT02671539 THOR ***	Did not meet 1° endpoint	2	rAAV2.REP1	University of Tubingen/ STZ eyetrial	**X**			
Choroideremia	NCT03507686 GEMINI	COMPLETED	2	timrepigene emparvovec (BIIB111/AAV2-REP1) (subretinal)	Biogen	**X**			
Choroideremia	NCT03496012 STAR	Did not meet 1° endpoint	3	timrepigene emparvovec (BIIB111/AAV2-REP1) (subretinal)	Biogen	**X**			
Choroideremia	NCT03584165 SOLSTICE	Enrolling by invitation	3	Long-term follow-up of AAV2-REP1 for CHM Patients	Biogen (previously NightstaRX)	**X (2°)**			

* ex-US, Japan, ** ex-US, China, *** ex-US, Germany; (2°) indicates a secondary endpoint.
